# Peripheral pulmonary tuberculoma diagnosed by targeted next-generation sequencing with manual mapping navigation: A case report

**DOI:** 10.1097/MD.0000000000044914

**Published:** 2025-10-03

**Authors:** Peng Zou, Yapei Cui, Ning Zhang, Ziming Huang, Hanxiao Ma, Huilin Gan

**Affiliations:** a Department of Respiratory and Critical Care Medicine, The Eighth Clinical Medical School of Guangzhou University of Chinese Medicine, Foshan, China; b Department of Respiratory and Critical Care Medicine, Foshan Hospital of Traditional Chinese Medicine, Foshan, China; c Department of Respiratory and Critical Care Medicine, The First Clinical Medical School of Guangzhou University of Chinese Medicine, Guangzhou, China; d Department of Respiratory and Critical Care Medicine, Clifford Hospital, Guangzhou, China.

**Keywords:** bronchoscopy, case report, manual mapping navigation, peripheral pulmonary lesions, targeted next-generation sequencing, tuberculoma

## Abstract

**Rationale:**

Pulmonary tuberculoma is a special type of tuberculosis, and tuberculoma located in the peripheral part of the lung tend to be confused with other pulmonary diseases such as peripheral lung cancer, so early diagnosis is challenging. Manual mapping navigation defined as freehand sketching of bronchial routes based on computed tomography (CT) images. Bronchoscopists use this technique to assist in alveolar lavage and histological acquisition of peripheral lung lesions. We report a case of a patient who was ultimately diagnosed with peripheral tuberculoma of the lung after bronchoscopic tissue biopsy and targeted next-generation sequencing (tNGS) by manual mapping navigation.

**Patient concerns:**

A 29-year-old man was hospitalized for a solid nodule of about 27 mm × 20 mm subpleural in the basal segment of the right lower lobe detected on CT screening.

**Diagnoses:**

The patient’s CT results suggest that the nature of the nodule is undetermined.

**Interventions:**

The patient underwent bronchoscopic biopsy, brushing, and bronchoalveolar lavage fluid for tNGS testing guided by manual mapping navigation, which was subsequently diagnosed as pulmonary tuberculoma.

**Outcomes:**

Pathological results suggested lymphocytic infiltration, interstitial fibrous tissue hyperplasia, and tuberculosis-causing mycobacterium complex was detected in bronchoalveolar lavage fluid by tNGS. Finally, the patient was transferred to a tuberculosis specialty hospital for ***antituberculosis treatment, and the CT scan was repeated to show the nodule was smaller.

**Lessons:**

Diagnosis of peripheral pulmonary lesions is challenging, however, the use of a manual mapping navigation system in combination with tNGS can help in the diagnosis of most lung lesions in institutions that cannot provide advanced bronchoscopy techniques.

## 1. Introduction

Tuberculosis, primarily affecting the lungs, is an infectious disease caused by the *Mycobacterium tuberculosis* complex.^[1]^ Pulmonary tuberculosis is one of the 10 leading causes of death worldwide,^[2]^ imposing a significant economic burden on society.^[3]^ Bacteriological examination plays a crucial role in the diagnosis of tuberculosis,^[4]^ however, traditional diagnostic tools such as acid-fast bacilli smear and mycobacterium culture, have the disadvantages of limited sensitivity and long time consumption.^[5]^ Targeted next-generation sequencing (tNGS) is a detection technique that combines ultra-multiplex PCR with high-throughput sequencing technology, demonstrating excellent diagnostic performance in mycobacterial infections.^[6]^

Pulmonary tuberculoma is a special type of tuberculosis that often lacks the characteristic clinical manifestations of the disease. In particular, tuberculomas located in the peripheral lungs can easily be confused with other lung diseases such as peripheral lung cancer.^[7]^ Studies have found that a considerable proportion of surgically removed lung nodules are benign, and among them, lung nodules diagnosed as tuberculosis after surgery are one of the most common types of benign nodules.^[8]^ For peripheral lung lesions, computed tomography (CT)-guided biopsy carries a high risk of complications,^[9]^ while conventional bronchoscopy has difficulty reaching the lesion site.^[10]^ Whereas electromagnetic bronchoscopy navigation (EBN) can significantly improve the sensitivity of the diagnosis of peripheral nodules, there are limitations such as high cost, time-consuming, and need for special equipment.^[11]^ Manual mapping navigation (MMN) guidance bronchoscopy is a technique that involves identifying the airways leading to the target lesion by reading thin-slice CT images and converting them into a schematic diagram related to the actual bronchoscopic vision, thereby precisely locating and collecting samples for needle biopsy.^[12]^ It has the advantages of low cost, short time consumption and easy implementation. We report a case of peripheral pulmonary tuberculoma diagnosed by manual mapping navigation-guided bronchoscopy technique combined with tNGS.

## 2. Case presentation

**Figure 1.**
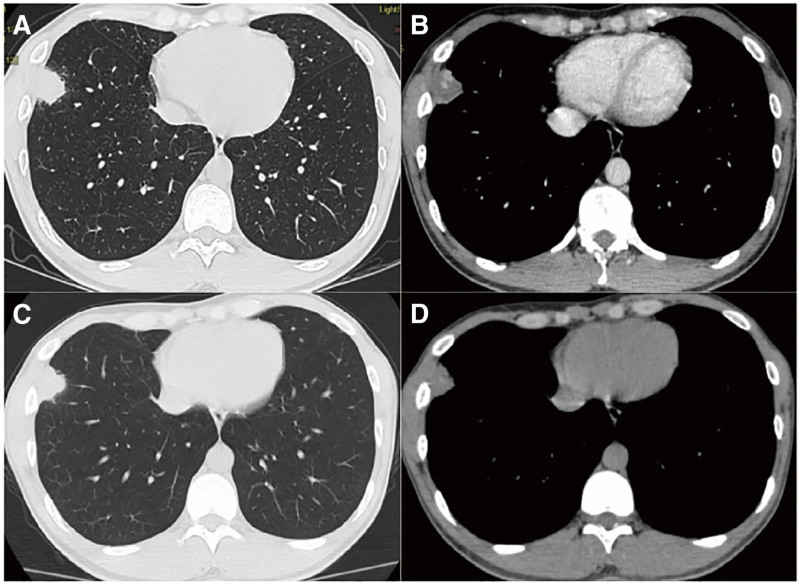
(A, B) Chest enhanced CT of the patient after admission, showing a solid nodule under the pleura of the anterior basal segment of the right lobe. (C, D) CT reexamination of the patient 1 month after antituberculosis treatment, showing that the original lesion has decreased in size.

A 29-year-old male was found to have a solid nodule subpleura of the anterior basal segment of the right lower lobe on a chest CT scan performed for incidental screening one and a half months before admission. Before admission, the patient underwent a chest CT scan again, which showed no significant changes in the lesion. Upon admission, the patient complained of mild cough and expectoration in the past year without any other discomfort. Further improvement of the chest CT enhanced scan showed solid nodule beneath the pleura of the anterior basal segment of the right lower lobe, approximately 27 mm × 20 mm in size, with lobulation sign and calcifications visible inside, and no enhancement was seen after enhancement (Fig. 1A, B). The patient denied a history of special diseases and having contact with animals. Laboratory findings: Tuberculosis-specific antigen-stimulated cytokine release assay showed a specific stimulated T-cell level of 311.79 pg/mL, indicating a positive(+) tuberculosis infection, and the tuberculin skin test was positive(+). Carcinoembryonic antigen and cryptococcal antigen, etc, were all normal. The lung nodule in this patient was considered an inflammatory nodule, and percutaneous lung biopsy was suggested but refused by the patient. We performed bronchoscopic lung biopsy under MMN for the patient, and also took bronchoalveolar lavage fluid (BALF) for tNGS.

**Figure 2.**
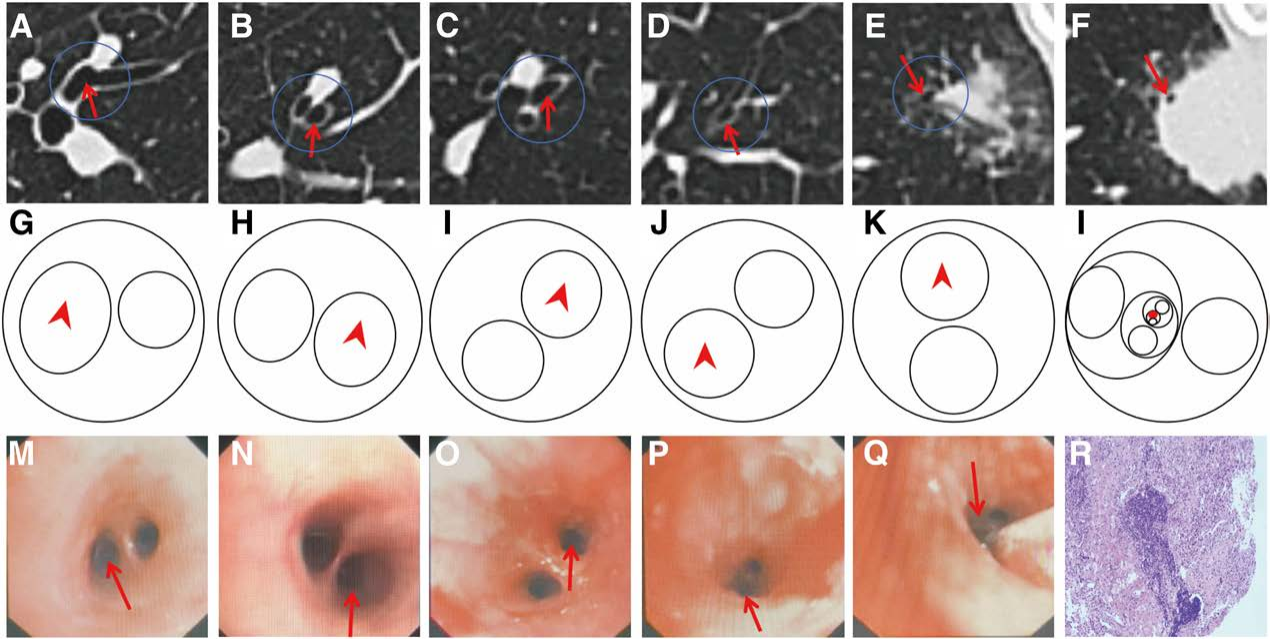
Illustrates the 5th to 9th generation bronchial opening maps targeting the lesion site, with the guide bronchus indicated. Panels (A–F) correspond to the computed tomography images of (G–L). Panels (G–K) represent the bronchial opening maps of the 5th, 6th, 7th, 8th, and 9th generations, respectively, where the lead bronchus leading to the target lesion is marked with a red arrow. Panel (L) summarizes all bronchial opening maps, with the red dot indicating the lesion located at the distal end of the 9th generation guide bronchus in the anterior basal segment of the right lower lobe. Panels (M–Q) correspond to the bronchoscopic images of (G–K), respectively. Panel (R) shows the cytopathological results of the lesion tissue biopsy.

Firstly, the bronchoscopic physician identifies the location of the target lesion on the CT image and then flips the chest CT image. Subsequently, by scrolling through thin-layer CT images, the bronchial route leading from the opening of the anterior basal segment of the right lower lobe to the target lesion is traced. A series of sketches are then manually drawn to depict the bronchial openings leading to the target lesion, marking the preceding bronchi step by step, and ultimately labeling the target lesion. After routine total airway examination, we inserted a ultrathin bronchoscope (UTB) (EB-530P, Japan Fujifilm) into the target lesion according to the route of the hand-drawn navigation map for biopsy, brushing, and alveolar lavage (Fig. 2).

BALF examination revealed Mycobacterium tuberculosis complex (283 sequences), acid-fast bacilli smear (1+). Biopsy pathology suggested that there was a lot of lymphocyte infiltration, interstitial fibrous tissue and focal histiocytic hyperplasia in the lung. Brush fluid suggested a few epithelial cells, histiocytes, and inflammatory cells. Combined with the history, etiological and pathological examinations, the diagnosis of tubculoma was confirmed, and the patient was referred to a tuberculosis specialty hospital for standardized antituberculosis treatment. After 1 month, the chest CT reexamined and the lesions were smaller than before (Fig. 1B, C).

## 3. Discussion

Navigation bronchoscopic techniques have a high diagnostic yield for peripheral pulmonary lesions (PPLs).^[13]^ Currently, several bronchoscope-guided techniques are available, virtual bronchoscopic navigation (VBN), EBN, radial endobronchial ultrasound and the combination these techniques.^[14]^ Although these techniques or procedures can significantly improve the sensitivity of bronchoscopy in the diagnosis of peripheral pulmonary lesions, they have drawbacks such as high cost, the need for special equipment, and the requirement for experienced bronchoscopists. In China, only a few large medical institutions have these advanced endobronchial intubation guided techniques.

MMN technology was first proposed by Kurimoto, which focused on the airway of interest and constructed a bronchoscopic roadmap by carefully analyzing airway bifurcation and its direction to the target lesion,^[15]^ so as to assist bronchoscopy. Kho et al^[16]^ reported a 98.9% navigation success rate, an 88.8% pathological diagnosis rate, and a 1% pneumothorax incidence rate when combining this method with R-EBUS for the diagnosis of PPLs. Other studies have also achieved high diagnostic rates by combining hand-drawn navigation techniques with the UTB-radial endobronchial ultrasound procedure for the diagnosis of PPLs.^[17]^

Compared to other bronchial navigation guidance techniques, the most significant advantage of MMN lies in its ability to reduce surgical time and costs. Studies have shown that the cost of electromagnetic navigation combined with UTB exceeds $2000, while the cost of standard thin-layer CT navigation combined with UTB and Lungpro virtual navigation combined with UTB ranges around $130–230.^[18]^ Research indicates that there is no significant difference in diagnostic rates for peripheral pulmonary nodules between MMN and VBN techniques. However, compared to the VBN group, the MMN group demonstrates significantly reduced planned path time, total surgical time, and operational costs (approximately $450 lower, including consumables).^[19]^ Experienced bronchoscopists can complete a MMN map in just 5 minutes.^[13]^ Furthermore, both VBN and EBN rely on CT and other imaging in the import system to generate navigation routes. Due to limited resolution of the peripheral airways of the lungs, this can lead to inaccurate navigation at the most critical final bifurcations.^[20]^ Bronchoscopists can mitigate this risk by carefully identifying small airways through repeated image review and drawing navigation maps.^[16]^ Lastly, regarding operator skill requirements, VBN operators need adequate training and experience to familiarize themselves with the software and techniques used to generate and navigate virtual models and make clinical decisions,^[11]^ whereas MMN has relatively lower operator requirements. However, training is still necessary for bronchoscopists using hand-drawn navigation, to familiarize them with CT analysis and its correlation with actual bronchoscopy views.^[16]^

Conventional pathogen diagnostics have limited sensitivity and specificity for tuberculoma diagnosis, and metagenomic next-generation sequencing (mNGS) can extract and sequence all genetic material fragments (DNA or RNA) from clinical samples, with the characteristics of hypothesis-free, bias-free, and comprehensive detection of microorganisms, which has a high sensitivity in pathogen detection.^[21]^ However, mNGS has several limitations, including high cost, significant human genetic interference, difficulty in interpreting results, and the inability to perform simultaneous DNA and RNA dual-processing detection.^[22]^ tNGS demonstrates similar overall performance to mNGS in pathogen identification,^[23]^ with additional advantages such as lower detection costs, easier workflow standardization, and the ability to simultaneously detect DNA and RNA pathogens.^[24]^ A recent retrospective study showed that the positive rate of mycobacteria detected by tNGS in bronchoalveolar lavage fluid was 93.8%, which was significantly higher than traditional methods such as acid-fast staining, culture, and pathology.^[25]^

Pulmonary nodules can be classified into benign (such as pulmonary tuberculosis, inflammatory pseudotumor and hemangioma) and malignant nodules based on their nature.^[26]^ The patient presented with mild cough and expectoration symptoms, positive T-SPOT results, and chest CT revealed a quasi-circular solid nodule located in the subpleural region of the lung. The nodule was calcified, lacked spiculation, and showed no pleural traction. There was no significant enhancement after the injection of contrast agent, which was consistent with the imaging features of pulmonary tuberculoma.^[27]^ Therefore, we considered the nodule in the lung periphery to be benign, with a high probability of being a tuberculoma. The lack of enhancement may reflect caseous necrosis with dystrophic calcification, differing from typical rim-enhancing tuberculomas.^[27]^ This underscores the imaging heterogeneity of pulmonary tuberculosis. Although we suggested performing imaging-guided percutaneous lung biopsy, the patient did not accept it due to concerns about related risks. Considering that the lesion was located in the periphery of the lung, it was difficult for conventional bronchoscopy to accurately reach the site. Thus, we performed bronchoscopic alveolar lavage, biopsy, and brushing under the guidance of MMN. Subsequent tNGS testing of BALF indicated *Mycobacterium tuberculosis* infection. Although definitive Langerhans giant cells were not observed in the histopathological examination of the lesion, there was a clear infiltration of lymphocytes and interstitial fibrous tissue hyperplasia, which were compatible with the pathological characteristics of pulmonary tuberculoma. Referring to the absence of Langerhans giant cells may be attributed to sampling limitations; thus tNGS served as a decisive diagnostic tool in this paucibacillary case. Finally, the patient was diagnosed with pulmonary tuberculoma and referred to a tuberculosis specialty hospital for standard antituberculosis treatment. After 1 month, the pulmonary nodule was found to be smaller compared to the previous assessment.

## 4. Conclusions

The diagnosis of PPLs is challenging and difficult to obtain by conventional bronchoscopy. This case report describes a patient with PPLs who was finally with pulmonary tuberculoma by bronchoscopy combined with tNGS guided by MMN. This approach can be used in most institutions unable to provide advanced choscopic technology and is inexpensive and highly accurate, which is worth further promotion.

## Author contributions

**Conceptualization:** Peng Zou.

**Data curation:** Peng Zou, Yapei Cui.

**Supervision:** Ziming Huang, Hanxiao Ma, Huilin Gan.

**Writing – review & editing:** Peng Zou.

**Writing – original draft:** Yapei Cui, Ning Zhang.
